# A novel circular invasion assay mimics *in vivo *invasive behavior of cancer cell lines and distinguishes single-cell motility *in vitro*

**DOI:** 10.1186/1471-2407-8-198

**Published:** 2008-07-14

**Authors:** Yoonseok Kam, Cherise Guess, Lourdes Estrada, Brandy Weidow, Vito Quaranta

**Affiliations:** 1 Department of Cancer Biology, Vanderbilt University School of Medicine, Nashville, TN, USA; 2 Department of Microbiology, Meharry Medical College, Nashville, TN, USA

## Abstract

**Background:**

Classical *in vitro *wound-healing assays and other techniques designed to study cell migration and invasion have been used for many years to elucidate the various mechanisms associated with metastasis. However, many of these methods are limited in their ability to achieve reproducible, quantitative results that translate well *in vivo*. Such techniques are also commonly unable to elucidate single-cell motility mechanisms, an important factor to be considered when studying dissemination. Therefore, we developed and applied a novel *in vitro *circular invasion assay (CIA) in order to bridge the translational gap between *in vitro *and *in vivo *findings, and to distinguish between different modes of invasion.

**Method:**

Our method is a modified version of a standard circular wound-healing assay with an added matrix barrier component (Matrigel™), which better mimics those physiological conditions present *in vivo*. We examined 3 cancer cell lines (MCF-7, SCOV-3, and MDA-MB-231), each with a different established degree of aggressiveness, to test our assay's ability to detect diverse levels of invasiveness. Percent wound closure (or invasion) was measured using time-lapse microscopy and advanced image analysis techniques. We also applied the CIA technique to DLD-1 cells in the presence of lysophosphatidic acid (LPA), a bioactive lipid that was recently shown to stimulate cancer cell colony dispersal into single migratory cells, in order to validate our method's ability to detect collective and individual motility.

**Results:**

CIA method was found to be highly reproducible, with negligible levels of variance measured. It successfully detected the anticipated low, moderate, and high levels of invasion that correspond to *in vivo *findings for cell lines tested. It also captured that DLD-1 cells exhibit individual migration upon LPA stimulation, and collective behavior in its absence.

**Conclusion:**

Given its ability to both determine pseudo-realistic invasive cell behavior *in vitro *and capture subtle differences in cell motility, we propose that our CIA method may shed some light on the cellular mechanisms underlying cancer invasion and deserves inclusion in further studies. The broad implication of this work is the development of a reproducible, quantifiable, high-resolution method that can be applied to various models, to include an unlimited number of parameters and/or agents that may influence invasion.

## Background

Cancer invasion from a primary tumor site is one of the most critical factors for determining cancer prognosis [1]. It is increasingly understood that changes in the adhesive and migratory capabilities of tumor cells, as well as the tumor microenvironment play critical roles in malignant tumor progression and invasion [2,3]. In order to successfully invade *in vivo*, metastatic cells must first permeate the basal lamina barrier, which is comprised of specialized matrix proteins, prior to entering neighboring tissue. During this process, it is believed that cells undergo changes in intercellular adhesiveness and motility, both of which may be important for invasion [4]. Given normal physiological conditions, such as with wound healing, cell motility is highly regulated. However, since cell motility appears to be aberrantly regulated in tumors, the question of what initiates and maintains this mechanism is highly relevant to the study of cancer progression [5,6].

Although repeatedly probed, the mechanisms that guide motility and infiltration of cells through the extracellular matrix (ECM) remain one of the least understood aspects of cell invasive behavior [7]. Achieving a better understanding of such mechanisms may assist in the development of anti-metastatic and anti-invasive therapies, potentially powerful tools in combating dissemination in cancer patients [8]. However, many existing methods designed to examine these mechanisms, such as classical wound-healing or invasion assays, are limited in their abilities to focus on realistic cell behavior in the presence of their microenvironment, particularly at the cellular level. Therefore, our goal in this study was to implement an updated, physiologically-relevant *in vitro *method in order to obtain a more reliable, detailed understanding of cancer cell dispersal and invasion *in vivo*.

### Wound-healing and classical assays

When skin is compromised, or wounded, the damaged epidermal edges migrate forward to cover the wound surface [9]. Fundamental to our understanding of wound-healing, is the knowledge that wound margins proliferate and migrate onto newly laid matrix in the wound gap [10]. Wound-healing assays have been carried out in tissue culture for many years to estimate the proliferation rates and migratory behavior associated with different cells and culture conditions [11]. Migration of cells can be conveniently studied *in vitro *by using these classical assays, whereby confluent epithelial cells are scratched with a tool such as a razor blade to remove a linear strip of cells from a monolayer. The filling or "healing" of the remaining "wounded" area is then observed using time-lapse microscopy [12,13]. Such a method can provide information regarding the behavior of those migratory cells that act to heal the inflicted wound, which indirectly provides additional information about cancer progression. However, as might be expected, when the initial "wounding" is not precisely controlled, this method is encumbered with problems of quantification and reproducibility [14].

When using the classical method, the wounded edges of the intact cell monolayer commonly retract on both sides of a crude, linear scratch. This suggests that many of the cells on the "wound" edge potentially lose their original morphology and function because they have been physically disrupted [15]. Additionally, since classical assays are produced using sharp objects, the migrating surface (dish or coverslip), which is often coated with extracellular protein(s) prior to monolayer growth, can also be damaged. In order to overcome these problems, a number of updated circular wound-healing assays (CWA) have been established that are less detrimental, and more standardized than the linear-scratch method [16,17]. The CWA method involves removal of a uniform, circular portion of cells from a confluent monolayer that is then allowed to heal towards itself. This technique can be further strengthened by minimizing the surface damage inflicted to the cells by employing the use of a soft silicon tip, in place of blunt trauma [16]. Although this method is adapted to provide a more standardized model of wound-healing and migration, and overcomes some of the obstacles associated with the traditional methods, it too is fairly simplistic and limits the depth of information that can be obtained and directly linked to *in vivo *results.

### Traditional invasion assays

**Figure 1:**
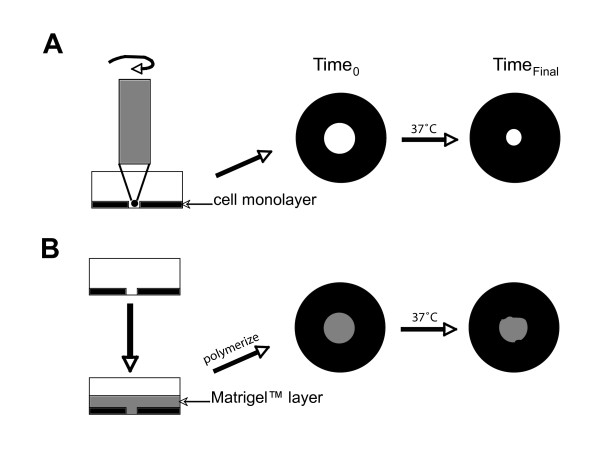
**Schemes of circular wound-healing (CWA) and circular invasion assays (CIA)**. For both methods, a stabilized, rotating, silicone-tipped drill press was used to create uniform, circular wounds in an intact confluent monolayer of cells (black ring) in a standard Petri dish. In contrast to the CWA method (A), the CIA technique (B) incorporates a Matrigel™ overlay (shown in gray), which acts as an extracellular matrix barrier that allows cells to invade more similarly to *in vivo *physiology. This added component enables detection of various degrees of cell invasion displayed over time, whereas a measurement of cell migration (or motility) is taken in its absence. Quantitation of wound closure is made by comparing the final wound size after a given incubation period (T_Final_) to the original wound size at 0 h (T_0_).

The invasive capacity of tumor cells and the mechanisms that control contrasting types of invasion are of critical importance in metastasis. Therefore, assays that determine this measurement in reproducible, quantitative terms can be extremely useful for probing these questions *in vitro*. The most common method currently employed to investigate invasive potential involves a modified Boyden chamber assay using a basement membrane matrix preparation (such as Matrigel™) as the ECM barrier and conditioned tissue culture media as a chemoattractant [18,19]. Although such a method is capable of supplying ample information about the collective migration of an entire population of cells, it fails to provide sufficient resolution for yielding precise, quantitative data for individual cell motility and invasion mechanisms. Therefore, in order to better distinguish between collective and individual movement of cells more clearly around "wounded" edges, and overcome the other problems associated with traditional assays, we have developed a novel circular invasion assay (CIA) modified from a previously established CWA technique designed by Watanabe and colleagues [16] (Figure 1A).

### Circular invasion assay (CIA)

For our method, a stabilized, rotating, silicone-tipped drill press was used to create eight uniform circular lesions in an intact monolayer, and the wound closure recorded and calculated for each using time-lapse microscopy and advanced image analysis. In contrast to the CWA method, the CIA technique utilizes an extracellular matrix component (Matrigel™) to build a pseudo-matrix barrier on the cell-free surface or "wounds" (Figure 1B). A number of two-dimensional *in vitro *invasion studies have previously shown that inclusion of this basement membrane component causes cells to behave similarly as they do *in vivo *[19-21]. Three-dimensional invasion assays have also been developed to include this reagent, or similar matrix components, due to an increased appreciation that their presence is necessary for demonstration of normal epithelial cell behavior [22,23]. Consequently, an obvious advantage to using our updated CIA method is the added environmental parameter (Matrigel™) that allows us to study invasion of cells in their microenvironment more accurately, while overcoming the need for advanced three-dimensional microscopy and analysis.

### Assessment of individual vs. collective invasion

In many physiological situations, such as with wound-healing or cancer metastasis, epithelium becomes motile under given stimulation [24]. In some instances, cells dissociate and individually explore their surroundings, whereas in other instances, the cells become collectively motile and enter the surrounding environment with their neighboring cells still intact. The difference between these behaviors has strong implications for our understanding of cancer-related dissemination [25].

As discussed in detail above, most classical *in vitro *migration/invasion assays reflect population dynamics, and are unable to provide information about individual cell behavior. Furthermore, *in vivo *tumor models often include "experimental" or "spontaneous" assays that assess the complete metastatic process, rather than focus its individual steps [26]. Recently, a number of both *in vitro *and *in vivo *methods have been developed which focus on illuminating the various mechanisms of motility at the single-cell level; however, the majority of these assays are performed using 3-D microscopy and fluorescent markers [27-29]. While these assays are often very informative, they can also be quite laborious and require advanced systems. In contrast, the CIA technique gives us the ability to dissect the metastatic process in 2-D into steps or types of events, such as collective or individual cell motility, potentially enabling us to build a more detailed model of cancer invasion.

## Methods

### Materials

All cell lines were obtained from American Type Culture Collection (ATCC, Rockville, MD). McCoy's 5a Modified Medium was also purchased from ATCC. L-α-lysophosphatidic acid (oleoyl sodium salt, LPA 18:1) was purchased from Avanti Polar Lipids (Alabaster, AL) and used at a concentration of 2 μM. Phosphate buffered saline solution (PBS), Dulbecco's Modified Eagle's Media (DMEM), fetal bovine serum (FBS), penicillin-streptomycin antibiotics, and L-glutamine were obtained from GIBCO BRL (Carlsbad, CA). Matrigel™ Matrix Growth Factor Reduced was purchased from BD Biosciences (San Jose, CA) and used at 50% concentration (in DMEM). The optimal concentrations of these reagents were determined in previously performed dose-dependent experiments, taking concentrations given in the literature as baseline values.

### Cell culture

We examined four cancer cell lines that have various established levels of invasiveness both *in vitro *and *in vivo *in order to test the applicability of our method to different stages of cancer progression. DLD-1 (CCL-221), a human colorectal adenocarcinoma cell line that is tumorigenic in nude mice [30,31], MCF-7 (HTB-22), a human mammary epithelial cell line found to be nonaggressive and noninvasive in mice [32], and MDA-MB-231 (HTB-26), a human mammary epithelial cell line found to be highly aggressive and known to rapidly progress to extensive and well-vascularized metastatic lesions [32,33], were routinely cultured and maintained in DMEM supplemented with 10% heat-inactivated FBS, 1% penicillin-streptomycin antibiotics, 1% L-glutamine, and kept in a humidified atmosphere of 5% CO_2 _at 37°C. SKOV-3 (HTB-77), a human ovarian epithelial cell line found to be moderately aggressive and tumorigenic in nude mice [34], was regularly maintained in McCoy's 5a Modified Medium also supplemented with 10% FBS, 1% antibiotics, 1% L-glutamine, and grown in the same incubator conditions.

All cell lines were seeded, in sterile conditions, at a density of 1.5–2 × 10^6 ^on polystyrene, 35-mm, tissue-culture treated Petri dishes (Falcon, Becton Dickinson Labware, Franklin Lakes, NJ) for 18–24 hours or until confluent. For indicated experiments (those involving LPA treatment), cells were serum-starved by incubation in DMEM in the absence of FBS for 18–24 h after monolayers reached confluence.

### Circular wound-healing assay (CWA)

Uniform, circular-shaped "wounds" (1.5 – 2 mm diameter; 8 per dish) were generated using a rotating drill press (Delta Shopmaster, Type 1, Model DP200) fit with a custom-shaped silicon tip (Home Depot; manually cut down to rounded shape with approximately 1.5 mm diameter and rounded bottom, using razor blade) as seen in Figure 1A. The optimal size and shape of the "wounds", their spacing, and other parameters were established in preliminary experiments (results not shown). The silicon tip was regularly washed with 70% ethanol between "wounding" of monolayers in individual dishes. Cell debris created by "wounding" was removed from each dish by manual pipetting, and intact cells were gently washed twice more with PBS. Two ml of growth media with 10% FBS was added to each dish for the remainder of incubation. For those experiments examining LPA effects, LPA or PBS in DMEM (2 ml total volume; without FBS) was directly applied into each dish and allowed to incubate for up to 24 h.

### Circular invasion assay (CIA)

For the novel CIA method, "wounds" were created as described above in the CWA method (8 per dish). Additionally, 50% Matrigel™ in growth media (600 μl total) was overlaid onto the "wounded" cell monolayer to create a matrix barrier against the cellular surface and allowed to polymerize for 15 min prior to imaging the original time point (Figure 1B). For those experiments examining LPA effects, LPA or PBS was added directly to Matrigel™ overlay prior to polymerization. Two ml of growth media with 10% FBS was added to each dish (LPA experiments used DMEM without FBS). "Wounded" monolayers, with fabricated matrix, were then incubated in a humidified atmosphere of 5% CO_2 _at 37°C for 24 h.

### Time-lapse microscopy

Time-lapse microscopy was conducted using a Zeiss Axiovert 200 M microscope (Zeiss, Thornwood, NY; 2.5× Plan NEOFLUR objective, NA 0.075; 10× Achroplan, NA 0.25, Ph1 objective) equipped with a Hamamatsu ORCA-ER CCD camera and temperature- and CO_2_-controlled chamber. Microscopy was under the control of OpenLab software (Improvision, Lexington, MA). At the beginning of each experiment (0 h), phase-contrast images were captured and microscopically accessed for standard, reproducible "wounds", with irregular outliers thrown out of the data set. Reflecting the precision of the method in creating consistent wounds by shape and size, this subpopulation of "unusable" wounds was negligible, as intra-operator variance was found to be < 3% (results not shown). Images of all "wounds" were then captured at regular time points for 24 h thereafter. The cell-free areas of each monolayer were distinguished from the surrounding intact cells by applying an automatic, software-defined threshold to each image, and pseudo-color applied to these areas using Adobe Photoshop 7.0 (Adobe Systems, Inc., San Jose, CA).

### Confocal microscopy analysis

A glass coverslip was coated by incubating in PBS containing 10 μg/ml of collagen I (C8919, Sigma, St. Louis, MO) overnight at 4°C. Circular wounds were made in the DLD-1 cell layer as previously described. Cells were further incubated for 5 hr at 37°C with or without 50% Matrigel™ overlay and fixed by 3.7% formaldehyde. Cortactin (green; lamellipodial marker) and actin (red; cytoskeletal marker) were visualized by immunofluorescence staining using anti-cortactin antibody 4F11 (Upstate Biotechnology Incorporated, Lake Placid, NY) and Alexafluor 568-phalloidin (Invitrogen). Confocal images were obtained with a Zeiss LSM-510 laser scanning confocal microscope equipped with a Plan-NEOFLUAR 40×/1.3 Oil DIC lens (Zeiss, Germany).

### Image analysis and quantitation

Time-lapse images were further processed using Java's *ImageJ *software (Wayne Rasband, National Institutes of Health, Bethesda, MD). Appropriate pairs of corresponding images were overlaid and compared to one another, to determine the difference between the pseudo-color applied areas measured from the original time point (0 h), to the final time point of interest (4, 6, 8, 12, or 24 h). This difference (in pixels) was then calculated and presented in terms of percent wound closure, or invasion measured.

### Statistical analysis

Each cell line was sampled at least 8 times for each method (N = 8–32; Power = 0.94–1.00), over the course of 10 days (N = 1–4 days per line). Wound repair data are referenced to time point 0 h, with results presented as mean percent wound closure (out of 100%) after a given period of time ± standard deviation. To avoid confounding problems with multiple analyses along the time-response curve, final differences were only analyzed at 4, 6, 8, 12, and 24 h. Differences between cell lines were examined using Student's t-tests, and were considered significant when P < 0.05. To further compare the two methods, post-hoc analysis (ANOVA) was performed for all parameters (method, treatment, time) using SPSS, Version 16 (SPSS Inc., Chicago, IL). Post-hoc power analyses were also performed for each set of experiments using G*Power 3 (E. Erdfelder, F. Faul and A. Buchner; University of Trier).

## Results

### Utility of CIA for assessment of differences in invasive capacity

The three cell lines used in the first set of experiments, MCF-7, SKOV-3, and MDA-MB-231, have been established to have varying degrees of invasiveness *in vivo*. In order to test our *in vitro *method's ability to translate to known physiological findings, we applied both the CWA and CIA techniques to these cell lines for comparison. Multiple, uniform wounds were created in all cell line monolayers with a custom-made silicon tip, as described in *Materials and Methods *(Figure 1). Starting areas of wounds (at 0 h) were highly and easily reproducible, with negligible levels of intra-operator variance experienced (< 3%; results not shown).

**Figure 2:**
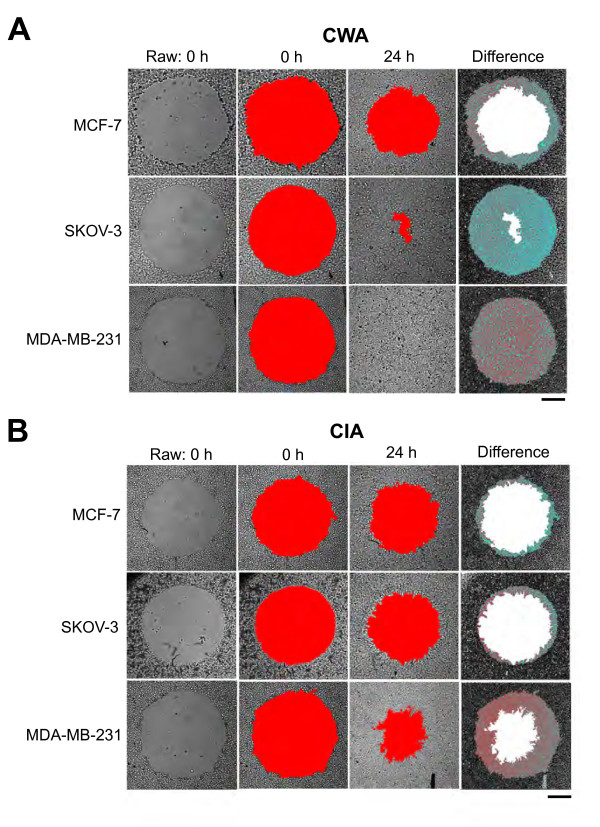
**Image analysis techniques and quantification method application**. Time-lapse images were obtained using a Zeiss Axiovert 200 M (2.5× Plan-NEOFLUOR objective, NA 0.075; scale bar = 500 μm) from application of the CWA (A) and CIA (B) methods. Each row of each panel represents one of three cell lines with various levels of aggressiveness (MCF-7, SKOV-3, and MDA-MB-231) captured at 0 h, 24 h, and the difference measured (in pixels) between the areas of those time points. The original, untouched wound for each cell line at 0 h is included in column 1 of each panel. The highlighted red regions (columns 2 and 3) indicate the pseudo-color applied (via ImageJ) to the wounded areas at both 0 h and 24 h, and the blue regions (column 4) indicate the differences (in area) obtained between those time points. Clearly, the Matrigel™ overlay has a considerable effect on wound closure of these cell lines.

Figures 2A and 2B represent the image analysis techniques and quantification method that was applied to time-lapse acquired images in order to obtain results for both the standard CWA and novel CIA techniques, respectively. The first two columns of each block of images show "wounded" monolayers from each of the three cell lines tested, MCF-7, SKOV-3, and MDA-MB-231, at time point 0 h (unprocessed raw images and pseudo-color applied, respectively). Notice that original wounds are comparable in size and shape, stressing the reproducibility of our method, which involves a semi-automated process of wounding cells using a standard carving tip. Column 3 includes the "healed" wounds after a 24 h incubation period in the presence (2B) or absence (2A) of Matrigel™. Pseudo-color (red) was applied to the open, cell-free areas of each image (using an automated threshold function applied by Adobe Photoshop), in order to establish a percent wound closure measurement for each time point. Finally, column 4 was created by overlaying the image from the final time point measured (24 h) over the original wound (0 h) in order to create the difference in area (shown in blue) between the snapshots. This area represents the final calculated percent wound closure obtained for each cell line at each time point. As evident by the unequal areas (red and blue pseudo-colors) applied to each image across the different cell lines, it is apparent that each cell line tested healed (and invaded) at different rates. It is also to be noted that when comparing the wound-closure obtained from the different methods, we see that those wounds incubated in the presence of Matrigel™ (2B) heal more slowly than those in its absence (2A). This may be due, in part, to the need of proliferating cells to actively degrade the added matrix barrier prior to invasion, or due to overall constraint (more discussion later).

**Figure 3:**
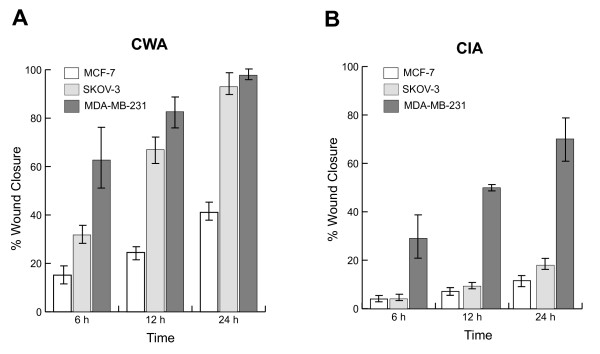
**Various cancer cell lines and degrees of invasiveness can be accessed by CIA**. Wound closure was measured for each cell line [MCF-7 (nonaggressive, noninvasive), SKOV-3 (moderately aggressive and invasive), and MDA-MB-231 (highly aggressive and invasive)] and presented as the mean ± standard deviation at 6, 12, and 24 h using both the CWA (A) and the updated CIA techniques (B). In the absence of Matrigel™, all cell lines exhibited significant levels of closure at each time point, which is contrary to expected levels of invasiveness (according to literature). In contrast, the CIA method detected the various levels of invasiveness of each cell line, in a manner that represents established levels found *in vivo*. Namely, MCF-7 and SKOV-3 invaded similarly at 6 and 12 h, but were significantly different (P < 0.05) from one another after 24 h. Furthermore, MDA-MB-231 invaded significantly more (P < 0.001) than the other cell lines at all time points.

Figure 3 is a side-by-side comparison of the quantitative results obtained from each assay, which further contrasts the two methods' abilities to distinguish between the different invasive properties of these cell lines *in vitro*. Figures 3A and 3B represent the percent wound closure and invasiveness (respectively) exhibited by the cell lines tested at multiple time points (6, 12, and 24 h), in the absence or presence of Matrigel™, respectively. Using the classical CWA method (Fig. 3A), MCF-7, the non-invasive, least aggressive cell line, exhibited the lowest degree of wound closure with no matrix present (15.15 ± 7.29, 24.50 ± 4.83, and 41.08 ± 6.86%, after 6, 12, and 24 hours, respectively). SKOV-3, the moderately invasive line, showed an intermediate-to-high level of closure (31.75 ± 8. 29, 66.98 ± 12.77, and 92.99 ± 9.89%). And MDA-MB-231, the most aggressive, invasive cell line, had the highest degree of wound closure at all time points measured (49.61 ± 11.14, 76.33 ± 9.48, and 96.91 ± 4.53%). While the CWA technique is capable of picking up variations in migration across the cell lines, it does not reflect the true separation of measurement expected for each on the basis of their known *in vivo *behavior. Namely, the MCF-7 wounds healed considerably at all time points, which is surprising given its characterization as a nonaggressive, noninvasive line [32]. Additionally, the separation between the SKOV-3 and MDA-MB-231 cells was not significant after 24 h (P > 0.05), which is contrary to the trends exhibited by these cells in previous studies *in vivo *[32-34].

In contrast, the CIA technique obtained results that were sensitive to the differences in *in vivo *invasive behavior of the cell lines (Figure 3B). With the addition of the Matrigel™ barrier, MCF-7 and SKOV-3 cells invaded similarly at both 6 h (4.91 ± 2.75% and 4.70 ± 2.58%, respectively) and 12 h (8.71 ± 4.07% and 10.66 ± 3.89%, respectively) time points, but were significantly different after 24 h (14.09 ± 7.88% and 20.47 ± 6.72%, respectively). Additionally, MDA-MB-231 invaded significantly (P < 0.001) more than the other cell lines at all time points measured (27.40 ± 5.09, 47.06 ± 7.64, and 66.05 ± 6.95%), as expected. From these figures, it is clear that as time progresses, the separation between levels of invasion further manifests itself, as often experienced in animal models [35]. Also, we noted that the variation and confidence levels associated with the CWA method were inferior to those of the CIA method (more discussion later). Clearly, our novel invasion assay has given us reproducible, plausible results for a variety of phenotypes, supported by previously published findings on these cell lines [32-34].

### Confocal microscopy analysis

**Figure 4:**
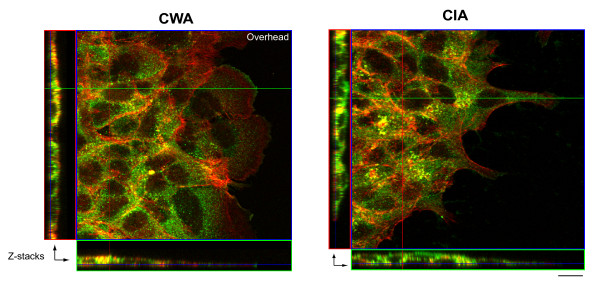
**Confocal micorscopy analysis**. Glass coverslips were coated by incubating in PBS containing 10 μg/ml of collagen I overnight at 4°C. Circular wounds were made in the DLD-1 cell layer as previously described in *Materials and Methods*. Cells were further incubated for 5 hr at 37°C in the presence or absence of Matrigel™ overlay and fixed by 3.7% formaldehyde. Cortactin (green; lamellipodial marker) and actin (red; cytoskeletal marker) were visualized by immunofluorescence staining using anti-cortactin antibody 4F11 and Alexafluor 568-phalloidin. Images were obtained with a Zeiss LSM-510 laser scanning confocal microscope equipped with a Plan-NEOFLUAR 40×/1.3 Oil DIC lens (scale bar = 10 μm). Lateral Z-stack images (captured from areas indicated by green, horizontal and red, vertical lines) indicate that in both assays, the cells remain at the plane level of the substrate (dish). In the CIA, cells seem to move along the interface of the dish and Matrigel™ overlay, rather than upwards into the gel. However, slight morphologic changes are observed between CWA and CIA methods at the same time point, indicating that Matrigel™ is providing some form of constraint on cells.

Figure 4 includes both overhead and stacked confocal microscopy images taken from the CWA and CIA techniques. The overhead images of closing wounds in DLD-1 cells display decisively different cell morphologies and shaped protrusions (lamellipodia) across methods. In the absence of Matrigel™(CWA), cells display a spread morphology and wide, fan-like lamellipodial protrusions, a morphology consistent with a migratory phenotype. In contrast, in the presence of the overlay, cells appear to be less spread and display thinner protrusions, perhaps more consistent with an invasive morphology. Additionally, there is an apparent higher concentration of cortactin on the dorsal cell side, presumably in direct contact with Matrigel™, suggesting that the cytoskeleton is organized differently in cells undergoing migration in the CIA vs. the CWA method. Furthermore, comparison of stacked images (taken from areas indicated by green, horizontal and red, vertical lines; CWA, 37 slices (16.01 μm); CIA, 23 slices (9.78 μm)) suggests that cells remain at the plane level of the substrate for both methods, but those cells plated with Matrigel™ apparently achieve a slightly greater depth (suggesting some interaction with overlay). While more in-depth studies are necessary to characterize these distinctions, these results support the notion that the CWA and CIA assay cell migratory abilities in different ways.

### CIA distinguishes between collective and individual cell motility

Epithelial cells generally proliferate in tightly packed colonies when seeded on plastic or glass under normal culture conditions. However, our lab recently reported that the addition of lysophosphatidic acid (LPA) to these cells induced dispersal of these colonies [36]. LPA is a bioactive lipid mainly synthesized by platelets that is found at micromolar range concentrations in plasma and serum, and has several effects on cells [37]. Relevant to this paper, this agent has been reported to cause dissociation of cell contact, leading to changes in morphology and behavior that spurs individual motility [36]. We introduced LPA in our CIA method in order to determine whether this assay can also be used to distinguish and quantify individual versus collective cell motility. Recently, these two kinds of cell motility have been proposed to play distinct roles in cancer invasion [25,38].

**Figure 5:**
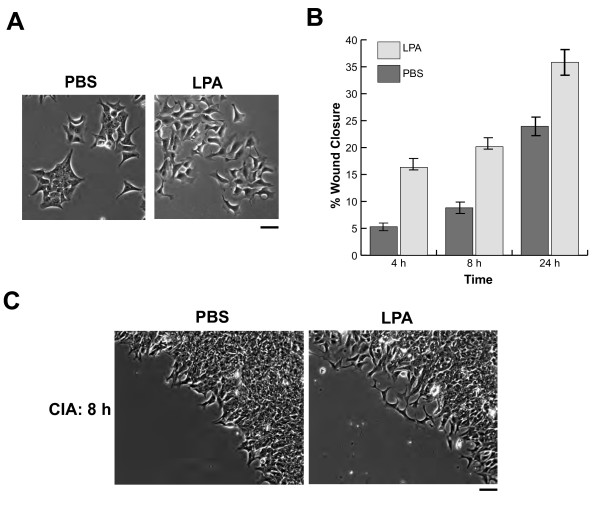
**LPA induces dispersal of DLD-1 cancer colonies**. Wounds were inflicted as described in *Materials and Methods *and images were obtained using a Zeiss Axiovert 200 M microscope (10× Achroplan objective, NA 0.25, Ph 1; scale bar = 50 μm). (A) DLD-1 cells undergo morphological changes and individually disperse upon exposure to LPA, compared to treatment with PBS. (B) Mean percent wound closure ± standard deviation measured in the presence or absence of LPA using the CIA method. (C) Images of PBS or LPA pretreated, "wounded" areas after 8 h of incubation. These results indicate that LPA both influences cell morphology (spurs individual behavior) and stimulates overall wound closure, compared to PBS treated cells (collective behavior).

Figure 5A includes images of DLD-1 cells (100×) in the presence or absence of LPA. We chose this cell line because it was previously shown to be stimulated by LPA [36]. These results show that LPA treatment promotes dissolution of DLD-1 colonies into individual cells, whereas PBS had little effect on cell dissociation and cells remained in direct contact with their neighboring cells. This figure also illustrates the typical cell morphological changes brought on by treatment with LPA, namely the onset of "ginkgo leaf-shaped" cells with increased lamellipodia formation and cell-cell dissociation with tails, as previously reported [36]. Results for these experiments were obtained using the same quantification method previously described using the other cell lines. The concentration of LPA employed in our experiments was 2 μM, which is within range of physiological concentrations (0.1 to 10 μM/L) that are generally found in plasma or serum [37]. A requirement for the LPA dispersal response is that cells undergo serum-deprivation for 24 h prior to LPA addition [36]; in non-deprived colonies, LPA had no effect (results not shown).

Figure 5B is a summary of results obtained from DLD-1 cells, in the presence or absence of LPA at various times over the course of 24 h, using the CIA method. The addition of LPA treatment significantly (P < 0.001) enhanced invasion at all time points using this method (10.53 ± 0.91, 17.77 ± 1.76, and 29.62 ± 2.55 at 4, 8, and 24 h, respectively) compared to the PBS control (6.51 ± 0.74, 10.32 ± 1.00, and 22.98 ± 2.23). Also noteworthy is the fact that as incubation time is increased, the difference of rates of healing between LPA and PBS further manifests itself. No differences in DLD-1 motility were observed between the CWA and CIA techniques (not shown), presumably because cells had to be serum-starved in order to sensitize them to LPA [36].

Figure 5C includes images (100× magnified) of the invasive fronts of invading DLD-1 cells at 8 h in the presence and absence of LPA. This time point was chosen because the characteristic "LPA effect" is known to occur around this period. Results taken from the later time point (24 h) distinguishes between the different treatments, but loses some of the classic cell morphology expected, based on our previous findings. As evident by the magnified images of these cells given each treatment, DLD-1 cells migrate into the cell-free area as single cells in the presence of LPA, and collectively in its absence. Similar to cells seen in Fig. 4A, LPA stimulation induces the expected morphological changes that include the onset of "ginkgo leaf-shaped" cells with increased lamellipodia formation and cell-cell dissociation with tails. Clearly, the proposed CIA method is compatible with high-resolution imaging that detects morphological nuances of single cells and successfully differentiates between collective and individual invasion *in vitro*.

## Discussion and conclusion

We present a novel, *in vitro *circular invasion assay (CIA) technique that overcomes many of the limitations associated with traditional wound-healing and invasion methods. Building upon a standard CWA technique that uses a silicon-tipped drill press, we have added a Matrigel™ matrix barrier to the CIA method prior to incubation, in order to examine cellular invasion over time *in vitro *(Figure 1). This added parameter, in addition to the use of high-resolution microscopy and image analysis techniques, gives our technique a four-fold advantage over many traditional assays; the CIA method is: 1) reproducible, 2) quantifiable, 3) physiologically-relevant, and 4) able to distinguish between collective and individual cancer cell invasion.

Classical wound-healing methods suffer from a variety of problems. Since these assays are commonly produced using crude, sharp objects (i.e. pipet or razor blade), it is often difficult to remove "wounds" without damaging intact cells' original morphology and function [15], or without inflicting damage to ECM protein(s) that are commonly laid prior to cell seeding in dishes [16]. Our CIA technique overcomes these problems, at least in part, by creating wounds with a soft silicon tip in place of using blunt trauma, which leaves both ECM and cells intact (see Additional files 1 and 2). Furthermore, since a standard tip and automatic drill is used for creating "wounds", the original size, shape, and spacing of replicates was found to be highly reproducible, and the assay was easily quantified. Furthermore, the variance associated with the data from the CWA method was found to be significantly (P = 0.036) higher than that of our CIA technique (due to larger standard deviations). Perhaps this was due to the less constrained range of motility that is associated with the matrix-free CWA technique, compared to a more constrictive microenvironment presented to cells in the CIA method. Increasing incubation time during performance of CIA may supercede this separation of methods, which could allow cells adequate time to display more movement and intrinsically more deviation.

Other *in vitro *methods, commonly using microfabrication-based or electrical impedance techniques, have been adapted to overcome the obstacles associated with traditional wound-healing experiments [14,16,38,39]. However, they are usually too rigid in design and make it impractical to obtain realistic cell behavior measurements *in vitro *(i.e. migration and invasion) that is largely dependent upon providing appropriate environmental parameters similar to those *in vivo *[19-23]. Figures 2 and 3 clearly show the importance of inclusion of an ECM-like barrier, such as Matrigel™, in establishing realistic invasive potentials of different cells *in vitro*. Notably, the rates of wound-healing measured using the CIA method, in the presence of the Matrigel™ overlay, were significantly (P < 0.001) lower than those obtained from the traditional CWA method for the three cell lines tested. These contrasting side-by-side results suggest the importance of the CIA technique, given that the rate of invasion measured for each of the cell lines tested using this method corresponds closely with previously published *in vivo *findings [32-34].

This distinct separation of measurements obtained from the CWA and CIA methods may be due, in part, to the need of cells to degrade the surrounding barrier during the invasion process [40,41]. Preliminary experiments were performed (results not shown), which found that inhibition of cell invasion can be observed with the addition of a broad-spectrum MMP inhibitor (GM6001). We tested concentrations up to 20 μM (in line with previous literature) for this agent and saw some response, however a higher concentration of agent may be needed for significant effect to be seen in this model. In order to reach definitive conclusions about the mechanisms by which cells deal with the ECM in the CIA, more in-depth studies are required. Confocal microscopy images (Figure 4) suggest that cells move forward through Matrigel™ in the CIA method, but remain attached to substrate (underlying dish). Although cells were not embedded *within *the matrix *per se*, the addition of the Matrigel™ overlay clearly enables measurement of cell invasion *in vitro*. From these results, it is tempting to speculate that the distinct dorsal distribution of cortactin in the CIA method may reflect engagement with the overlaid matrix of actin-based cellular organelles, such as lamellipodia or invadopodia. More analyses are obviously necessary to address this point conclusively. Nonetheless, this observation further supports the utility of the CIA assay for studying features of cell migration and invasion that other assays do not cover.

The CIA technique also enables researchers to focus on invasion at the single-cell level (in contrast to population driven methods). Figure 5 clearly demonstrates that LPA significantly increases individual cell motility and invasion, compared to collective behavior in PBS. In theory, single cells have the potential to move through smaller spaces and to travel longer distances than cells constrained by the necessity to move collectively. Therefore, it could be argued that epithelial cells that migrate with their neighboring cells pose a smaller risk of forming metastases than do those individually migrating cells [42]. Alternatively, it has also been proposed that collectively motile groups of cells are more protected from harsh environmental factors that potentially kill individual cells [5]. Given this information, and that LPA has been reported to enhance both migration and invasion in a number of other *in vitro *studies [43-45], suggests that LPA-induced cell scattering and migration may play an essential role in cancer invasion. Regardless, the ability to distinguish between these different types of cell behavior is clearly important for in depth studies of invasion and dissemination. Figure 5C captures the ability of the CIA method to detect both collective and individual cell invasion, given appropriate stimulation. Again, this implies the versatility of this technique and its potential to be applied to a wide range of models.

The applications that could be addressed using this technique are many. Since a soft silicon tip is used for "wounding", a variety of ECM molecules can be laid prior to cell seeding without obstruction of this component; this enables more flexibility when choosing cell lines and other growth conditions for experimentation. Furthermore, the Matrigel™ overlay can be customized (both by manufacturer and in-house) to include a variety of agents and/or therapeutics that may inhibit or promote invasion, allowing researchers to easily incorporate their own molecules of interest. Similar to our own experiments, these studies can be set up to quantitate average levels of cell invasion or can focus on collective versus individual cell motility at the leading edge, given appropriate stimulation. Alternatively, using high-magnification, time-lapse microscopy and/or fluorescently labeled cells, this method could also be applied to studying dynamic cell invasion mechanisms such as formation of lamellipodial protrusions. We foresee using this model, or slightly adapted versions of it, to further probe cell line differences between wild-type and knockdown cell lines; this will provide us with a relative relationship between cells due to particular mutations. While, at the moment, the CIA is lower-throughput than standard Boyden invasion assays, and may provide less resolution than some 3-D techniques, it certainly fills a niche in available 2-D *in vitro *invasion assays and deserves consideration in future works.

## Abbreviations

2-D: two-dimensional; 3-D: three-dimensional; CIA: circular invasion assay; CWA: circular wound-healing assay; DMEM: Dulbecco's modified Eagle's media; ECM: extracellular matrix; FBS: fetal bovine serum; GM6001: galardin; LPA: lysophosphatidic acid; MMP: matrix metalloproteinase; PBS: phosphate buffered saline; TB: trypan Blue.

## Competing interests

The authors declare that they have no competing interests.

## Authors' contributions

YK performed all DLD-1 assays, contributed intellectual property, and assisted in manuscript preparation. CG performed all MCF-7, SKOV-3, and MDA-MB-231 assays, and assisted in manuscript preparation. LE participated in design and coordination of the study, contributed intellectual property, and assisted in manuscript preparation. BW performed all statistical analysis and assisted in manuscript preparation. VQ, the corresponding author, conceived of the study, participated in its design, and helped to draft the manuscript. All authors read and approved the final manuscript.

## Pre-publication history

The pre-publication history for this paper can be accessed here:



## Supplementary Material

Additional file 1 Classical wound-healing method versus CIA: Comparison of ECM and cell damage. After DLD-1 cells were grown to confluence on laminin-332 coated Petri dishes, wounds were created using either a standard pipet tip to manually scratch cells similarly to classical assays (top row), or a silicon-tipped drill for the CIA technique (bottom row). Post-wounding, the dishes were stained with anti-laminin-332 polyclonal antibody (2778; green) and actin (red), and imaged with a Zeiss Axiovert 200 M (10× Achroplan, NA 0.25, Ph1 objective; scale bar = 50 μM). Employing a classical scratch method, both the laminin-332 undercoat and cells were damaged when "wounding" with a pipet. In contrast, both components appear to be minimally affected and left intact, by application of the CIA technique. Click here for file

Additional file 2 **CIA: **Cell death and debris. (A) DLD-1 cells were grown to confluence overnight, wounded by a silicon-tipped drill press machine, stained with 0.2% Trypan Blue (TB) solution in DMEM for 5 min to detect disrupted (dead) cells and debris, washed once in PBS, and imaged using a Zeiss Axiovert 200 M (10× Achroplan, NA 0.25 objective; scale bar = 100 μM). (B) The monolayer was subsequently washed three times in PBS to remove additional debris, and again stained with TB. (C) Cells were incubated for an additional 16 h at 37°C and again stained with TB. Clearly, the wounding technique employed causes a minimal level of death and debris (stained blue; converted to gray-scale). Click here for file
