# Supplementary figures and images for: A novel circular invasion assay mimics in vivo invasive behavior of cancer cell lines and distinguishes single-cell motility in vitro
Source: BMC Cancer. 2008 Jul 14;8:198. doi: 10.1186/1471-2407-8-198 (PMC2491634; doi:10.1186/1471-2407-8-198)

**Laminin**

**Cells**

**Overlay**

**Pipet**

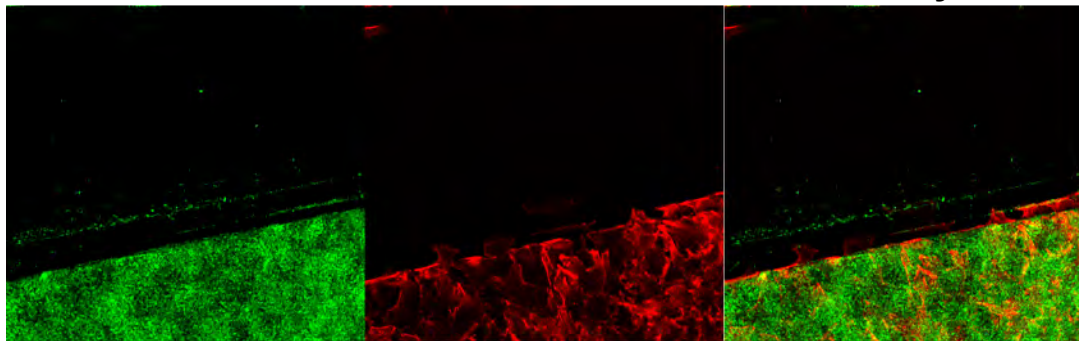

**CIA**

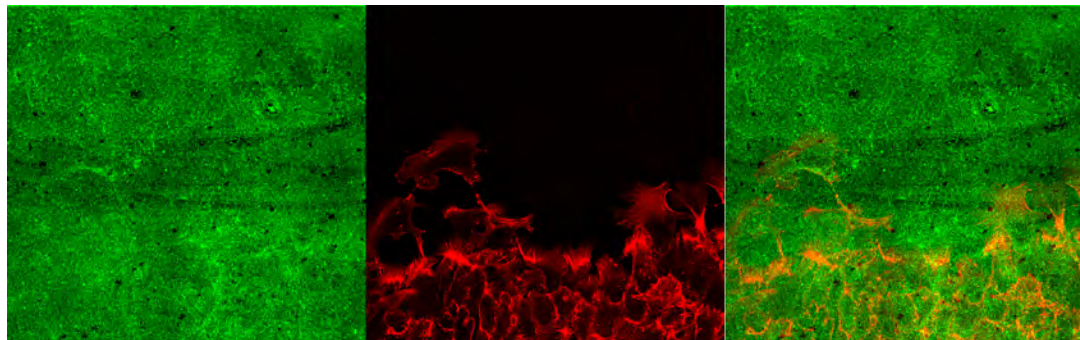

Supplement: Additional file 1 — Classical wound-healing method versus CIA: Comparison of ECM and cell damage. After DLD-1 cells were grown to confluence on laminin-332 coated Petri dishes, wounds were created using either a standard pipet tip to manually scratch cells similarly to classical assays (top row), or a silicon-tipped drill for the CIA technique (bottom row). Post-wounding, the dishes were stained with anti-laminin-332 polyclonal antibody (2778; green) and actin (red), and imaged with a Zeiss Axiovert 200 M (10× Achroplan, NA 0.25, Ph1 objective; scale bar = 50 μM). Employing a classical scratch method, both the laminin-332 undercoat and cells were damaged when "wounding" with a pipet. In contrast, both components appear to be minimally affected and left intact, by application of the CIA technique. [file 1471-2407-8-198-S1.pdf]

Trypan Blue  
Staining

Pre-wash

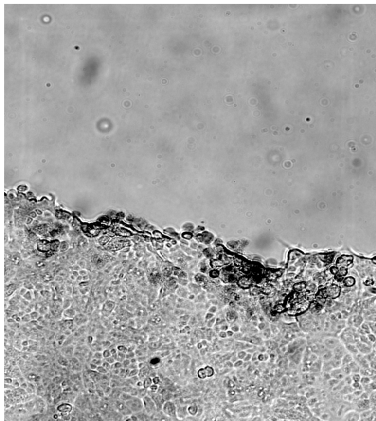

Post-wash

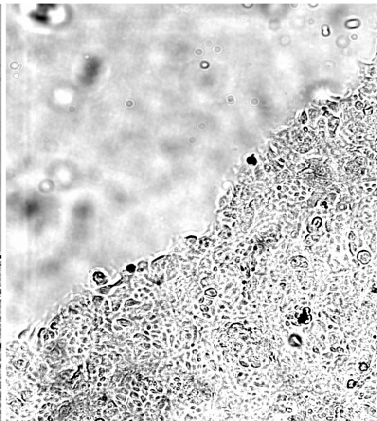

Post-incubation (16 h)

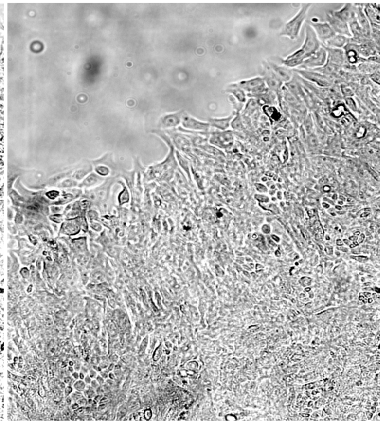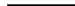

Supplement: Additional file 2 — CIA: Cell death and debris. (A) DLD-1 cells were grown to confluence overnight, wounded by a silicon-tipped drill press machine, stained with 0.2% Trypan Blue (TB) solution in DMEM for 5 min to detect disrupted (dead) cells and debris, washed once in PBS, and imaged using a Zeiss Axiovert 200 M (10× Achroplan, NA 0.25 objective; scale bar = 100 μM). (B) The monolayer was subsequently washed three times in PBS to remove additional debris, and again stained with TB. (C) Cells were incubated for an additional 16 h at 37°C and again stained with TB. Clearly, the wounding technique employed causes a minimal level of death and debris (stained blue; converted to gray-scale). [file 1471-2407-8-198-S2.pdf]
